# Barriers in Office-Based Opioid Treatment in Rural United States

**DOI:** 10.7759/cureus.73373

**Published:** 2024-11-10

**Authors:** Savitha K Satyasi, Christopher Stewart, Kaushal Parimi, Suporn Sukpraprut-Braaten, Nauman Ashraf

**Affiliations:** 1 Psychiatry, Freeman Health System, Joplin, USA; 2 Medical School, Kansas City University of Medicine and Biosciences, Joplin, USA; 3 Statistics, Washington University at St. Louis, St. Louis, USA; 4 Graduate Medical Education, Kansas City University of Medicine and Biosciences, Joplin, USA

**Keywords:** addiction medicine, medication based treatment for opioid use disorder, opioid use disorder, quality improvement research, rural access to health, rural health services

## Abstract

Background

The opioid crisis has severely impacted health outcomes in the United States, particularly in rural areas, where barriers to medication-based treatment for opioid use disorder (OUD) persist. Although medication-assisted treatment (MAT) for OUD is effective, access remains limited, especially in these communities.

Aim

This study identifies and examines barriers to accessing office-based OUD treatment in rural areas of the United States.

Methods

We conducted a cross-sectional survey of 49 adults with OUD treated at an outpatient facility in rural Missouri. The survey assessed familiarity with OUD medications, barriers to accessing treatment, difficulties finding providers, and support systems. Data were analyzed using descriptive statistics and chi-square tests to compare accessibility and emotional barriers.

Results

The primary barriers identified were related to accessibility (54%), including costs, insurance, clinic hours, and transportation, significantly outweighing emotional barriers like stigma or lack of support (26%). Accessibility barriers were notably higher than cases reporting no barriers (p<0.01) and higher than those who reported emotional barriers (p<.05). This highlights the need for improved infrastructure and support.

Conclusion

Cost, insurance, clinic location, and limited clinic hours are significant obstacles to OUD treatment in rural areas. Addressing these barriers through strategies like expanded clinic hours, telehealth, transportation assistance, and physician education is essential to improving access to care for OUD patients in rural settings.

## Introduction

The opioid epidemic has contributed to reducing life expectancy and had a harmful impact on health in the United States, especially in rural areas [1,2]. The most recent national survey estimates that at least 2.7 million people in the United States have opioid use disorders (OUD) [3]. Commonly used opioids include prescribed medications such as hydromorphone and codeine, as well as illicit substances such as fentanyl and heroin [4]. Additional treatment and research are needed to address this epidemic [5]. One such treatment that holds the potential to increase health outcomes in these patients is medication-assisted treatment. 

Medication-assisted treatment (MAT) of OUD involves using medications to treat individuals with an OUD [5]. Research shows that the use of medications to treat OUD more than doubles abstinence outcomes compared to a placebo or treatment with no medication [5]. The most commonly used treatments include methadone, buprenorphine, and extended-release naltrexone [5]. Access to (MAT) and the availability of educated, qualified physicians to provide this care are limited, particularly in rural communities. This study addresses barriers to evidence-based care for treating opioid addictions in the rural United States. 

While effective treatment is available for opioid users, there is a significant lack of access to this care throughout the United States, particularly in rural areas [2]. Key barriers to care identified in the literature include stigma, treatment experiences, fear, logistical issues, and a lack of patient education [6,7,8]. In addition, physicians in rural areas reported higher levels of bias towards patients with OUD than their urban counterparts, adding an additional barrier to OUD treatment [8]. Individuals in rural areas are also more likely to struggle with polysubstance abuse in addition to OUD, highlighting the importance of target prevention and treatment interventions in the rural United States [9]. 

One key barrier for OUD patients is the shortage of qualified addiction medicine specialists and psychiatrists in rural areas [10]. Less than 4% of licensed physicians are approved to offer services in medication-assisted treatment for OUD [11]. Further, less than 40% of patients with OUD are able to access MAT for their condition [11]. This gap is even more pronounced in rural areas, which have both higher rates of OUD and fewer qualified physicians. Identifying and addressing these barriers is crucial to improving health outcomes for OUD patients in the rural United States. This study aims to identify and address barriers faced by OUD patients living in rural areas to enhance health outcomes. 

## Materials and methods

Study design, settings, and IRB

This study is a cross-sectional survey conducted at the Ozark Center, an outpatient facility in Joplin, Missouri, between August and October 2023. Ethical approval was granted by the Freeman Health System Institutional Review Board, and informed consent was obtained from all participants. The study was classified as low-risk, and patient confidentiality was maintained through data de-identification.

Study population

The study population consisted of adults aged 18-65 who were receiving treatment for opioid use disorder (OUD) at the Suboxone outpatient facility. Inclusion criteria required participants to be in outpatient care for OUD. Exclusion criteria included pregnant women, incarcerated individuals, and other vulnerable groups to maintain consistency and reduce potential biases.

Study definition

Access to care was defined by geographic and logistical factors, including the location of the clinic, transportation availability, and clinic operating hours. Emotional barriers were defined as factors such as lack of support from family and friends, anxiety, and perceived stigma around seeking treatment.

Study tool

The survey instrument was designed to assess patients' familiarity with OUD medications, barriers to accessing treatment, and support systems for treatment adherence (Appendices). Questionnaire development was based on common barriers identified in clinical practice. Although the survey was not based on any previous survey, survey questions were rigorously reviewed by board-certified psychiatrists and addiction medicine specialists. The survey was administered digitally, and responses were recorded anonymously.

Outcome variables

Primary outcome variables included the frequency and types of barriers encountered in accessing OUD treatment. Barriers were classified as accessibility-related (location of clinic, transportation to clinic, and clinic hours) or emotional (lack of support, anxiety, and a feeling of being judged). Additional data collected included OUD treatment options. 

Data management and statistics

Descriptive statistics were used to summarize demographic data and barrier frequencies. Chi-square tests were employed to compare accessibility and emotional barriers as well as accessibility barriers against the absence of barriers. Analyses were conducted using Microsoft Excel (Microsoft Corporation, Redmond, USA).

## Results

A total of 49 people participated in the study. The average age is 39 (±10.2) years old. The patient demographic information can be seen in Table 1. 

**Table 1:** Demographics of the Participants

Category	Demographic	N	Mean (SD) or Percent
Patient Age (years)		49	39 (10.2)
Patient Race (percent)	White	37	75.5%
	Native American	2	4.1%
	Other	3	6.1%
	Not Listed	7	14.3%
Education (percent)	College Degree	5	10.2%
	Associates Degree	4	8.1%
	Trade School	2	4.1%
	Some College	5	10.2%
	High School/General Educational Development (GED)	21	42.9%
	Some High School	6	12.2%
	None	2	4.2%
	Not listed	4	8.1%

**Figure 1:**
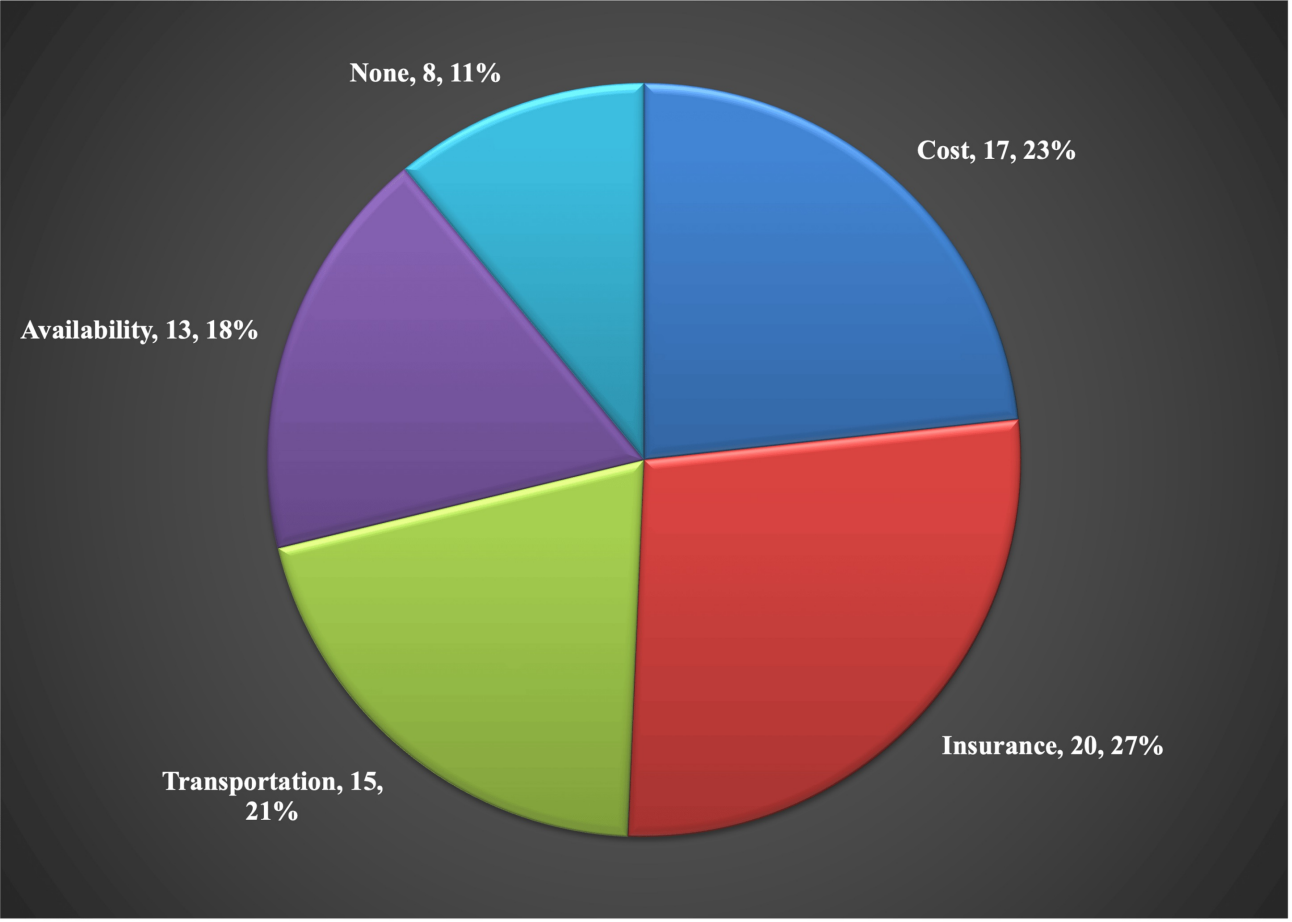
Most Common Barriers to Opioid Use Disorder Treatment Opioid Use Disorder (OUD). Data presented as Barrier, Number of patients, Percent of total barriers reported.

**Figure 2:**
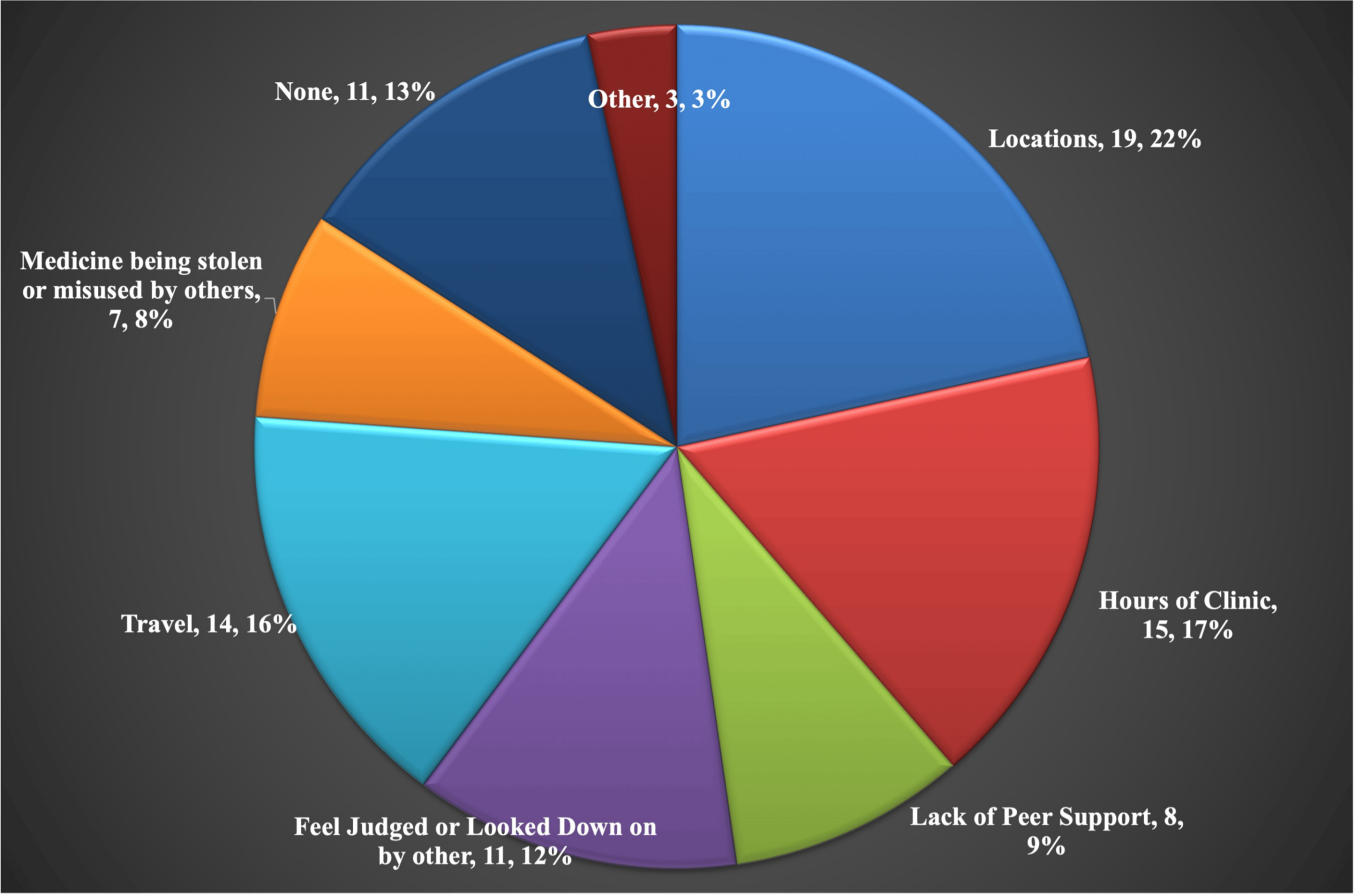
Factors that Decrease Availability to Opioid Use Disorder Treatment Opioid Use Disorder (OUD). Data presented as Factor, Number of patients, Percent of total barriers reported.

**Figure 3:**
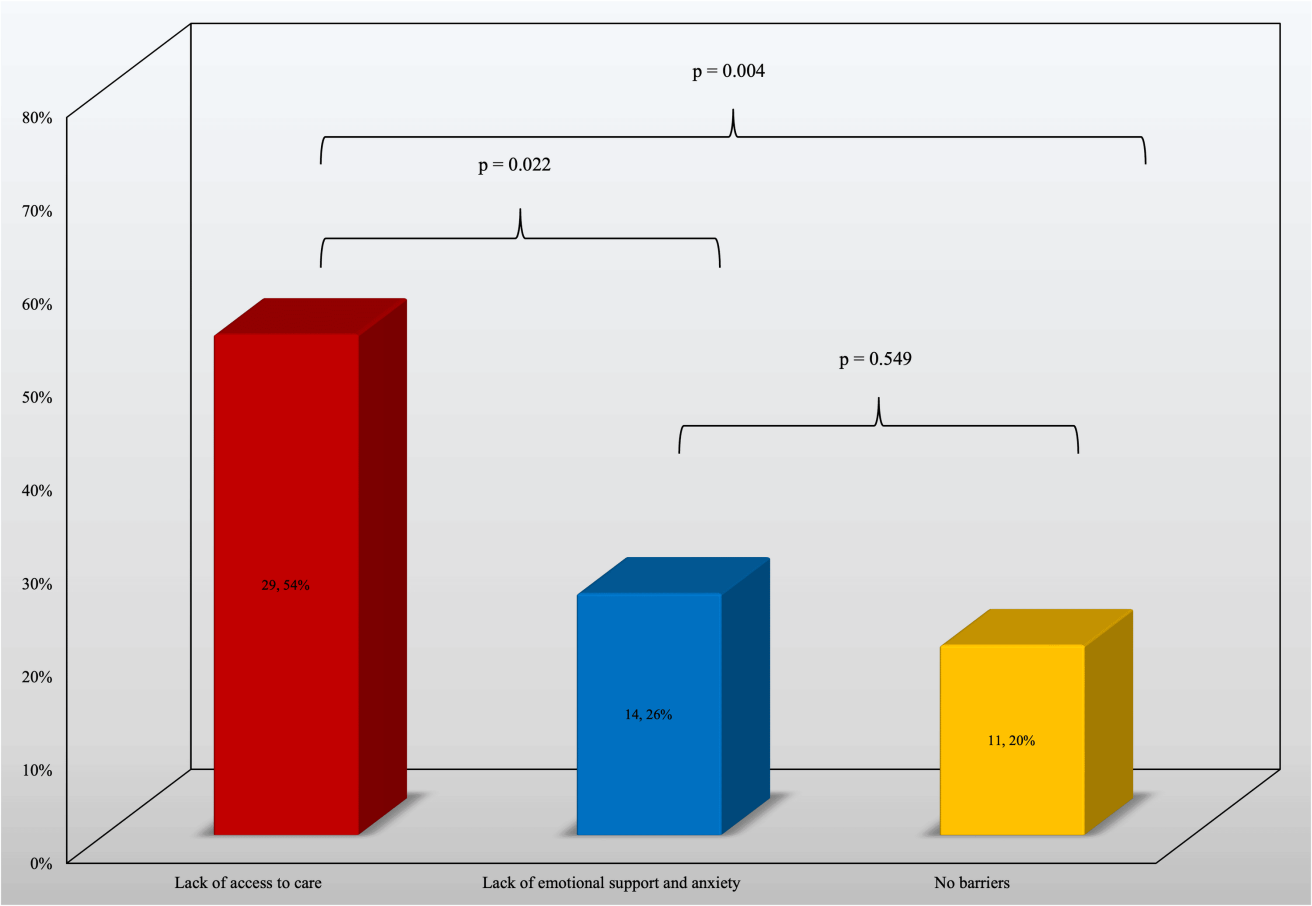
Comparison of Barriers to Accessing Office-Based Opioid Treatment Opioid Use Disorder (OUD). Data listed as number, Percent (%). Statistical program R version 4.4.1 (R Foundation for Statistical Computing, Vienna, Austria) was used to compute the chi-square test comparing the number of patients who reported the barrier reasons.

The primary barriers preventing patients from accessing medication for opioid use disorder are cost-related factors, including expenses and insurance issues (57%). These findings are summarized in Figure 1. Note that in Figures 1-3, the total number of reported barriers exceeds the number of participants. This is because patients reported all barriers faced, not just a single barrier. The percentages in Figure 1 and Figure 2 show the percentage of total reported barriers/factors. 

The most common factors that were found to decrease the availability of OUD treatment were the location of services (38%), hours of clinic (30%), and travel services to clinics (28%). Note in Figure 2 that the number of individuals is greater than the number of individuals as on the survey individuals could select more than one factor. These findings, along with other factors are found in Figure 2. 

Results also showed a significant difference of 28% (p=.022) when comparing access to care and emotional barriers. This identifies accessibility barriers as the primary barrier to OUD treatment. A significant difference was also found between a lack of access to care and no barriers to access (43%, p=.004) indicating there are barriers. This highlights access to care as the main barrier to OUD treatment (Figure 3). 

## Discussion

Cost and insurance analysis

While treatment with opioid use disorder (OUD) has been successfully administered using medications, such treatment requires access to qualified medical professionals. Commonly used medications include methadone, buprenorphine, and extended-release naltrexone, all approved by the Food and Drug Administration (FDA) and considered the safest treatments for OUD for treating OUD [12]. Further, medication treatment of OUD has shown a decreased risk of mortality in OUD patients [13]. This underscores the importance of understanding and ensuring access to OUD treatments. 

As shown in Figure 1, cost and insurance are the leading barriers to OUD treatment in the rural United States. Identifying these root causes is crucial for developing strategies to reach these patients. This finding highlights the need for insurance policies to cover OUD medications and for patient education on accessing these treatments. The Medicaid expansion in 2008, which covers adults with an income under 138% of the federal poverty level, has increased access to OUD care [14]. However, about 14% of insurance plans still do not cover any OUD medications [14]. Additionally, the costs of treatments like methadone, buprenorphine, and extended-release naltrexone can be prohibitive, reaching up to $200,000 without insurance [15]. Despite existing measures, cost remains the primary barrier to accessing OUD treatment.

Accessibility vs emotional barriers

Another significant finding of this study is the comparison between accessibility to OUD treatment and emotional barriers to OUD treatment (Figure 3). Accessibility barriers include clinic location, transportation, and clinic hours, while non-cost-related barriers include lack of support, anxiety, and fear of judgment. Patients could select multiple barriers, resulting in more patients included in Figure 3 than the number of participants. There was a significant difference in the proportion of patients facing cost-related versus both emotional barriers (28%, p=0.022) and no barriers at all (34%, p=.004)

This finding is noteworthy because it suggests that the primary reason individuals do not receive OUD care is not due to social stigma or judgment but rather due to a lack of access to resources. Understanding these limitations enables physicians to offer more empathetic and effective care. It also highlights the need for more addiction medicine specialists, particularly in rural areas. Increasing the number of specialists in these areas can enhance patient access to OUD treatment. Additional strategies to improve access include providing transportation to clinics, expanding telehealth services, and using mobile clinics. Educating physicians about OUD treatment is also crucial, as many do not prescribe OUD medications due to stigma or lack of knowledge [4]. Moreover, MAT for OUD is not standardized within psychiatric care [4]. Increasing physician education can help address physical barriers and improve care for individuals with OUD. Implementing these strategies can address the key reasons for the lack of access to OUD treatment and help combat the OUD crisis in rural United States.

Limitations, bias, and areas of future study

One limitation of this study is the small sample size and the fact that all data were collected from a single clinic in rural America. Additionally, the lack of diversity in our patient demographics is a limitation. Different regions have varying needs and populations, which may not be fully reflected in our findings. While the results may be applicable to other rural addiction clinics, they may not be as relevant to urban populations. Moreover, the data were self-reported, which introduces potential bias. Since participants were already receiving care for opioid addiction, they may have been less likely to report barriers such as feeling judged or unsupported. Future studies should examine the impact of physician education, especially in psychiatry and family medicine, and replicate this research in other locations to validate and expand the findings.

## Conclusions

Primary barriers to OUD treatment in rural areas are the clinic location, travel services, and clinic hours. This study helped us identify these barriers in order to develop strategies and solutions to overcome the barriers and increase access to OUD treatment. OUD is a real problem in the rural United States, and in order to increase access to treatment that can help these patients, we must understand the barriers they face. Based on the barriers identified, providing transportation, expanding the clinic hours, expanding access to telehealth and mobile clinics, reducing stigma and discrimination, providing physician education, and providing peer support and counseling can help overcome barriers to OUD treatment to decrease the mortality rate due to OUD in rural areas of the United States. 

## Appendices

Survey:

1. Age?

2. Highest Level of Education?

3. Race?

4. Are you familiar with the different types of medication that can help you with your opioid problem?

     Methadone: yes/no

     Buprenorphine (Suboxone): yes/no

     Naltrexone: yes/no

5. What are the main reasons that make it hard for you to get the medication you need? Choose all that apply

     Cost

     Insurance

     Transportation

     Availability

     None

6. Have you had trouble finding a doctor or a clinic that can prescribe you medication for your opioid problem?

Yes

No

7. Do you feel comfortable with talking to your doctor or other health care providers about your opioid problem and your treatment options?

Yes

No

8. Do your family, friends, and community encourage you to seek and continue treatment?

Yes

No

9. Do you access information and resources about opioid problems and treatment options?

Yes

No

10. Do you cope with the stigma that some people may have towards people who take medication for their opioid problems?

Yes

No

11. Are you satisfied with the quality and effectiveness of the medication you are taking?

Yes

No

12. What are some of the things that would make it harder for you to get and stay on treatment for your opioid problem? Choose all that apply

     Locations

     Hours of clinic

     Lack of peer support

     Feel judged or looked down on

     Travel

     Medicine being stolen

     None

     Other

     My work hours

     Feel anxiety
